# A Rare Case Report of Flecainide-Induced Left Bundle Branch Block (LBBB) and Transient Cardiomyopathy

**DOI:** 10.7759/cureus.37184

**Published:** 2023-04-05

**Authors:** Sukhjinder Chauhan, Desiree Morris, Mina Bhatnagar, Pinak Shah, Dhiraj D Narula

**Affiliations:** 1 Internal Medicine, Mountainview Hospital, Las Vegas, USA; 2 Electrophysiology, Mountainview Hospital, Las Vegas, USA

**Keywords:** flecainide adverse effects, pacemaker induced cardiomyopathy, congestive heart failure due to flecainide, chf, left bundle branch block, flecainide possible lbbb, flecainide toxicity, flecainide, transient nonischemic reversible cardiomyopathy, cardiomyopathy

## Abstract

Flecainide is an antiarrhythmic agent that has been reported to have numerous cardiotoxic effects, including the development of arrhythmias and the reduction of left ventricular ejection fraction (LVEF). However, it is not commonly reported as a cause for left bundle branch block and cardiomyopathy.

In this case report, we present the case of a 67-year-old female patient who developed transient cardiomyopathy and left bundle branch block (LBBB) secondary to flecainide therapy. The patient's condition improved upon cessation of flecainide.

## Introduction

Cardiomyopathies are a heterogeneous group of conditions characterized by muscular and/or electrical dysfunction of the heart [1]. These conditions are an important cause of progressive heart failure and cardiovascular death and can be classified as primary (i.e., genetic, acquired, or mixed) or secondary (i.e., toxic, inflammatory, or infiltrative). Major types include dilated cardiomyopathy, restrictive cardiomyopathy, hypertrophic cardiomyopathy (HCM), and arrhythmogenic right ventricular cardiomyopathy [1,2]. Drug-induced cardiomyopathy is a potentially reversible form of cardiomyopathy, which may occur due to exposure to several medications such as chemotherapeutic agents (e.g., anthracyclines, trastuzumab), antiretroviral drugs, and antipsychotics [3].

In this report, we present a case of transient cardiomyopathy with a left bundle branch block (LBBB) in a 67-year-old female who was taking flecainide, a commonly used antiarrhythmic agent. Based on the patient’s presentation and exclusion of all other possible diagnoses, it was concluded that the cardiomyopathy was caused by flecainide.

## Case presentation

A 67-year-old Caucasian female, with a past medical history of sick sinus syndrome status post a dual chamber pacemaker implantation about six months ago, paroxysmal atrial fibrillation (PAF), and hypertension, was brought to the hospital by the emergency medical services (EMS) for pleuritic chest pain. The pain was substernal, non-radiating, worsened upon exertion, and did not improve with rest. She also reported orthopnea, paroxysmal nocturnal dyspnea, and bilateral lower extremity edema that had been gradually worsening. She had been diagnosed with paroxysmal atrial fibrillation (PAF) after the pacemaker implant and was subsequently started on flecainide 150 mg BID. She denied any past medical history of coronary artery disease (CAD), congestive heart failure (CHF), and illicit drug abuse. Her other home medications included cyclobenzaprine, trazodone, buspirone, and ibuprofen. 

**Figure 1:**
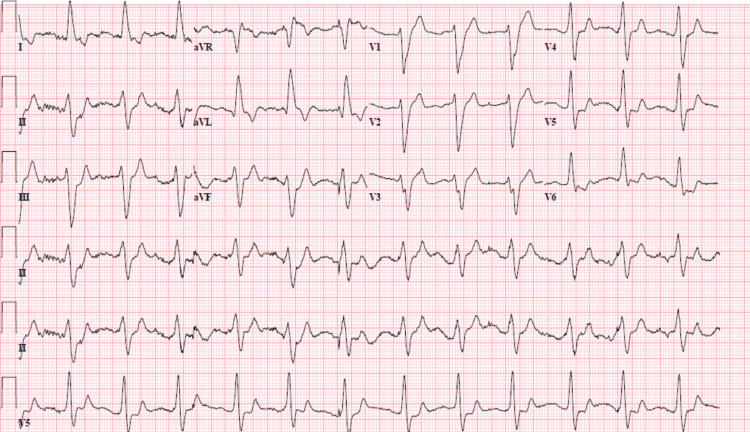
12-lead EKG demonstrated a new left bundle branch block (LBBB) with left axis deviation and a wide QRS complex of 194 milliseconds on admission. EKG: Electrocardiogram, LBBB: Left bundle branch block

On admission, the patient was afebrile. She had a blood pressure of 164/70 mmHg and was hypoxic, requiring supplemental oxygen. Complete blood count (CBC) was unremarkable. Comprehensive metabolic panel (CMP) was significant for a serum potassium level of 5.6 mEq/L, blood urea nitrogen (BUN) of 19 mg/dL, creatinine of 1.35 mg/dL, N-terminal pro-b-type natriuretic peptide (NT-proBNP) of 8,767 pg/mL, and high sensitivity troponin levels of 38 ng/mL. The 12-lead electrocardiogram (EKG) shown in Figure 1 demonstrated a new LBBB with left axis deviation and a wide QRS complex of 194 milliseconds.

**Figure 2:**
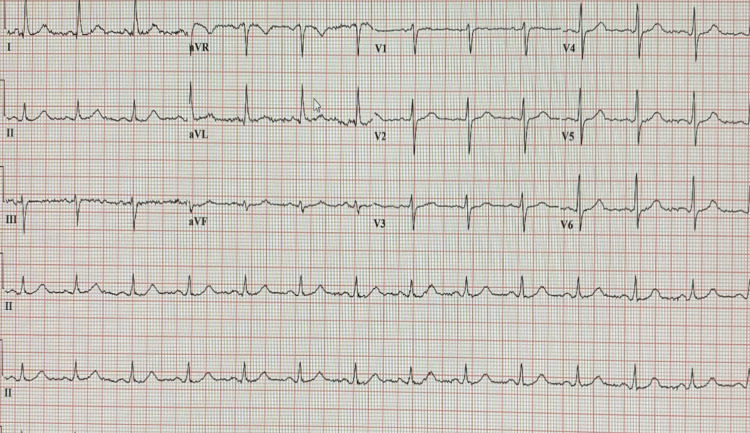
EKG from 2013 was unremarkable and did not show LBBB EKG: Electrocardiogram, LBBB: Left bundle branch block

A prior EKG from 2013 (Figure 2) was unremarkable and did not show LBBB.

**Figure 3:**
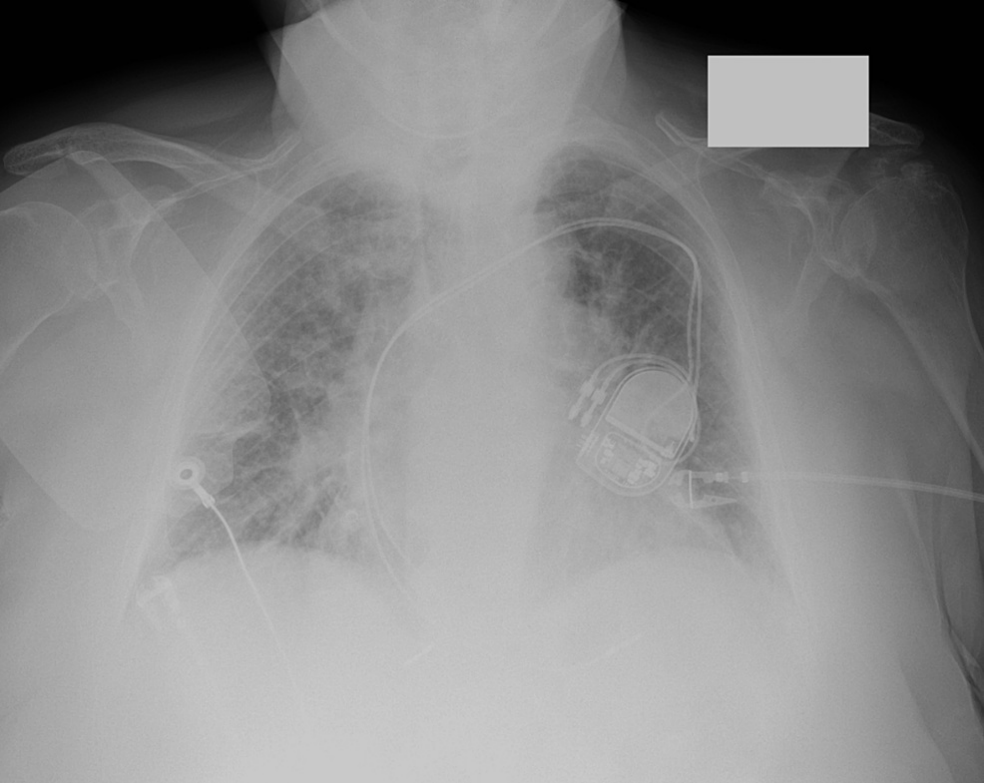
Chest radiography demonstrated bilateral diffuse interstitial opacities and interstitial pulmonary edema with central pulmonary vascular engorgement, cardiomegaly, and a dual-chamber pacemaker. CXR: Chest X-ray

Chest radiography demonstrated bilateral diffuse interstitial opacities and interstitial pulmonary edema with central pulmonary vascular engorgement, cardiomegaly, and a dual-chamber pacemaker (Figure 3).

Computed tomography pulmonary angiography (CTPA) was negative for pulmonary embolism. Transthoracic echocardiography (TTE) demonstrated a left ventricular ejection fraction (LVEF) of 35-40% with multiple wall motion abnormalities, including apical wall dyskinesis and akinesis of the apical anterior, mid anteroseptal, and apical septal walls, along with grade 2 diastolic dysfunction (Video 1).

**Video 1:** TTE demonstrated an LVEF of 35-40% with multiple wall motion abnormalities, including apical wall dyskinesis and akinesis of the apical anterior, mid anteroseptal, and apical septal walls, along with grade 2 diastolic dysfunction TTE: Transthoracic echocardiography, LVEF: Left ventricular ejection fraction

**Figure 4:**
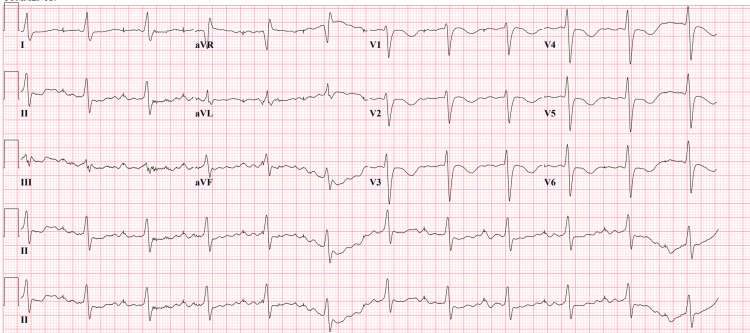
12-Lead EKG showing QRS complex duration improved from 194 milliseconds to 150 milliseconds after 24 hours following flecainide cessation EKG: Electrocardiogram

**Figure 5:**
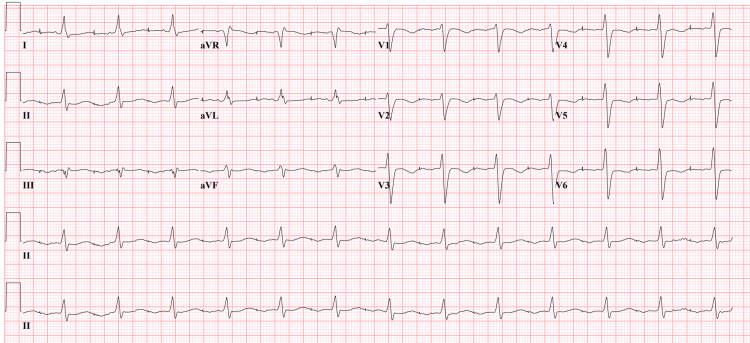
12-Lead EKG showing QRS complex duration improved from 150 milliseconds to 142 milliseconds after 42 hours following flecainide cessation. EKG: Electrocardiograms

**Figure 6:**
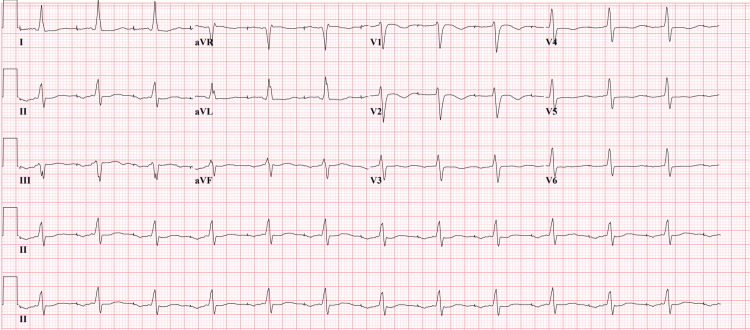
12-lead EKG showing QRS complex duration improved from 142 milliseconds to 122 milliseconds after more than 72 hours following flecainide cessation EKG: Electrocardiogram

The patient was admitted to the intensive care unit (ICU), was placed on bilevel-positive airway pressure (BiPAP), and was treated with diuretics for pulmonary edema. The patient responded well to diuretics and was successfully weaned off from BiPAP to a low-flow nasal cannula. A heparin drip was initiated for possible non-ST elevation myocardial infarction (N-STEMI) ischemic cardiomyopathy and paroxysmal atrial fibrillation (PAF). During this hospitalization, the patient's home medication, flecainide, was withheld due to a suspected flecainide-induced new-onset left bundle branch block and transient cardiomyopathy, indicated by the initial EKG, which demonstrated a prolonged QRS of 194 milliseconds. Follow-up electrocardiograms were conducted at 24, 42, and 72 hours after cessation of flecainide. EKGs showed a gradual improvement in QRS duration from 194 milliseconds to 152 milliseconds after 24 hours (Figure 4), 142 milliseconds after 40 hours (Figure 5), and 122 milliseconds after 72 hours (Figure 6). 

With an improvement in the respiratory status, cardiac catheterization was performed on day 3, which showed no evidence of coronary artery disease. Data from the pacemaker interrogation revealed that the dual-chamber pacemaker was 81% atrial paced and 25% ventricular paced with no significant atrial fibrillation, and the longest episode of arrhythmia was noted to be 8 seconds long. Transthoracic echocardiography (TTE) was repeated on the 4th day shown in Video 2, demonstrating an improvement in the LVEF from 35% to 50%.

**Video 2:** Transthoracic echocardiography (TTE) was repeated on the fourth day, which showed an improvement in LVEF from 35% to 50% TTE: Transthoracic echocardiography, LVEF: Left ventricular ejection fraction

The cardiac electrophysiology (EPS) team was consulted for further management. The pacemaker was reprogrammed to a longer atrioventricular (AV) delay of 280/250 milliseconds with prolongation up to 400 milliseconds, to allow intrinsic AV conduction. The patient's home medication, flecainide, which was initially held, was discontinued before discharge due to a suspected flecainide-induced new-onset left bundle branch block and transient cardiomyopathy.

On discharge, guideline-directed medical therapy (GDMT) for HF with improved ejection fraction was initiated with low-dose lisinopril 2.5 mg daily, and apixaban 5.0 mg BID was prescribed for thromboembolism prevention in the setting of PAF. The patient was advised to follow up with outpatient cardiology and the electrophysiologist within one week for further management. 

## Discussion

Flecainide is an oral class Ic antiarrhythmic agent, which is used for the prevention and treatment of supraventricular arrhythmias such as atrial fibrillation (AF), atrioventricular reentrant tachycardia (AVRT), atrioventricular nodal re-entrant tachycardia (AVNRT), and Wolff-Parkinson-White (WPW) syndrome. It may also be used for the treatment of life-threatening ventricular arrhythmias, refractory to other treatment options [4,5].

Flecainide works by blocking the open-state fast inward sodium channels, thus markedly depressing phase 0 of the action potential. It also inhibits IKr channels, delaying the potassium rectifier current. This results in the prolongation of the duration of action potential in both ventricular and atrial muscle fibers. The drug also inhibits ryanodine receptor 2 (RyR2), which reduces calcium release from the sarcoplasmic reticulum, thus decreasing arrhythmogenic calcium current. In addition, flecainide decreases the inflow of sodium and calcium ions in myocardial cells. This causes a negative inotropic effect, thus reducing the cardiac output and stroke volume [4,6]. Flecainide is absorbed almost completely from the gastrointestinal tract and reaches peak serum concentration in one to three hours. It is hepatically metabolized via the CYP450 system and then excreted in the urine. Its half-life ranges from 12-27 hours but may be prolonged in patients with heart failure, liver disease, or renal disease [6].

Flecainide has a narrow therapeutic index (0.2-1.0 mcg/mL). Therefore, small differences in dose or blood concentration can result in life-threatening adverse drug reactions. Flecainide toxicity can cause an excess blockade of the cardiac sodium channels, which can lead to delayed conduction, negative inotropy, and fatal arrhythmias, such as ventricular tachycardia and ventricular fibrillation [7]. Toxicity may be precipitated in patients with liver and/or renal failure secondary to decreased drug metabolization and clearance. Electrolyte abnormalities, such as hyponatremia, may contribute to cardiac toxicity by enhancing the inhibitory effect of flecainide on cardiac sodium channels [8,9]. Other non-cardiac adverse effects may include dizziness, visual disturbances, abdominal pain, and constipation [4]. Due to its negative inotropic effects, the drug is contraindicated in patients with congestive heart failure, coronary artery disease, cardiomyopathy, and left ventricular ejection fraction (LVEF) of less than 30%. In this patient population, it has been shown to cause a significant reduction in the stroke volume index and LVEF, while increasing the right atrial pressure and pulmonary capillary wedge pressure (PCWP) [9,10]. 

Finally, flecainide has been associated with a proarrhythmic effect, which can lead to the development of atrial flutter or ventricular tachyarrhythmia. For instance, it has the potential to convert atrial fibrillation to atrial flutter with 1:1 atrioventricular (AV) conduction, resulting in rapid tachycardia and a heart rate of more than 200 beats per minute. Drugs that block the AV node, such as beta-blockers and calcium channel blockers, should be used concurrently to lower this risk [4,10]. In the Cardiac Arrhythmia Suppression Trial (CAST), an increased rate of mortality and nonfatal cardiac arrest was observed in patients with a history of myocardial infarction. Based on the results of this study, the apparent proarrhythmic effect of flecainide is believed to be caused by its use in patients with structural abnormalities of the heart. Therefore, flecainide is contraindicated in patients with structural heart disease [11,12].

Our patient presented with CHF and new-onset LBBB. The differential diagnosis included ischemic cardiomyopathy (based on the new finding of LBBB with elevated serum troponin levels), arrhythmia-induced cardiomyopathy (given the patient’s history of sick sinus syndrome and atrial fibrillation), pacing-induced cardiomyopathy (due to the patient’s recent history of pacemaker implantation), and drug-induced cardiomyopathy (based on the patient’s use of flecainide). Ischemic cardiomyopathy was ruled out, as cardiac catheterization showed no signs of coronary artery disease in our patient. Arrhythmia-induced cardiomyopathy was excluded, as the data retrieved upon pacemaker interrogation demonstrated that the longest arrhythmia episode was only eight seconds long and that there were no episodes of atrial fibrillation.

Next, the possibility of pacing-induced cardiomyopathy (PICM) was considered. PICM is a rare complication of pacemaker therapy that is characterized by a reduction in LVEF and an increase in the end-systolic volume and wall stress, mainly due to ventricular dyssynchrony where the ventricles beat out of rhythm with each other [13,14]. A study on right ventricle (RV) pacing-induced LBBB has reported that when the ventricular lead is positioned in the apical region of the RV, it may result in an Intraventricular conduction delay (IVCD) that presents electrocardiographically as an LBBB pattern [15]. Treatment of PICM may involve adjusting the pacemaker settings or switching to a different type of device. However, it is generally recommended to minimize right ventricular pacing as much as possible and to use atrial pacing instead, as this type of pacing is less likely to cause pacemaker-induced cardiomyopathy [13,16]. PICM was excluded in our patient upon pacemaker interrogation, as it demonstrated a normal functioning pacemaker with 81% atrial pacing and 25% ventricular pacing.

As previously mentioned, flecainide toxicity can cause a widening of the QRS interval as an adverse effect. This is due to the drug's ability to slow down the conduction velocity of electrical signals in the ventricles, which can lead to a prolonged QRS complex on an EKG. A widened QRS interval of more than 122 milliseconds is typically observed [6]. The half-life of flecainide ranges from 12 to 27 hours, and in patients with heart failure, it can last up to 70 hours [6]. In our patient's case, there was a gradual improvement in QRS duration from 194 milliseconds on the initial EKG to 122 milliseconds on the EKG obtained after 72 hours of flecainide cessation. This suggests that the prolonged QRS duration was likely due to flecainide toxicity. While volume optimization could have aided in the improvement of ejection fraction (EF), the patient's LVEF improvement from 35% to 50%, however, in combination with the gradual improvement in QRS duration after flecainide discontinuation, without any significant alterations in pacemaker settings, provides additional evidence for the diagnosis of flecainide-induced transient cardiomyopathy.

Several studies have also shown that LBBB can be caused by flecainide toxicity secondary to a decrease in the electrical conduction in the left ventricular myocardium [12,17,18]. Our patient's EKG, shown in Figure 1, was remarkable for a QRS duration of 194 milliseconds with a new onset of left bundle branch block, as shown in Figure 1. All these findings supported the diagnosis of flecainide toxicity in our patient.

## Conclusions

Cardiomyopathy due to chronic exposure to medically prescribed drugs with cardiotoxic effects, such as anti-cancer, antiretroviral, and antipsychotic drugs, is common. However, the diagnosis remains particularly challenging for other drugs that are not commonly associated with the development of drug-induced cardiomyopathy.

Flecainide is an antiarrhythmic agent that is not a common cause of cardiomyopathy; however, the proarrhythmic effects, such as QRS, widening as an adverse effect of this medication are well-documented. Therefore, physicians should be aware of the potential cardiotoxicity of this drug and perform routine monitoring with EKG and patients should follow up with cardiology closely for the early identification of any adverse effects. Discontinuation of flecainide therapy and the use of alternative medications should be considered.
